# The Use of Wavelength Modulated Raman Spectroscopy in Label-Free Identification of T Lymphocyte Subsets, Natural Killer Cells and Dendritic Cells

**DOI:** 10.1371/journal.pone.0125158

**Published:** 2015-05-20

**Authors:** Mingzhou Chen, Naomi McReynolds, Elaine C. Campbell, Michael Mazilu, João Barbosa, Kishan Dholakia, Simon J. Powis

**Affiliations:** 1 SUPA, School of Physics and Astronomy, University of St Andrews, Fife, KY16 9SS, United Kingdom; 2 School of Medicine, University of St Andrews, Fife, KY16 9TF, United Kingdom; 3 Instituto de Engenharia Biomedica, 4150–180, Porto, Portugal; Hannover Medical University, GERMANY

## Abstract

Determining the identity of cells of the immune system usually involves destructive fixation and chemical staining, or labeling with fluorescently labeled antibodies recognising specific cell surface markers. Completely label-free identification would be a significant advantage in conditions where untouched cells are a priority. We demonstrate here the use of Wavelength Modulated Raman Spectroscopy, to achieve label-free identification of purified, unfixed and untouched populations of major immune cell subsets isolated from healthy human donors. Using this technique we have been able to distinguish between CD4^+^ T lymphocytes, CD8^+^ T lymphocytes and CD56^+^ Natural Killer cells at specificities of up to 96%. Additionally, we have been able to distinguish between CD303^+^ plasmacytoid and CD1c^+^ myeloid dendritic cell subsets, the key initiator and regulatory cells of many immune responses. This demonstrates the ability to identify unperturbed cells of the immune system, and opens novel opportunities to analyse immunological systems and to develop fully label-free diagnostic technologies.

## Introduction

The mammalian immune system comprises distinct bone marrow-derived cell types that interact to provide protection against an extensive array of potential pathogens including bacteria, viruses, fungi and parasites. Monitoring changes in the numbers of these cells in human blood can indicate the presence of inflammation and infection.

In humans the population of lymphocytes known as T cells can be divided into two main groups based upon their expression of CD4 and CD8 cell surface proteins[1]. CD4^+^ T cells usually function through the secretion of bioactive cytokines [2], whereas CD8^+^ T cells are typically known as cytotoxic T cells, which can directly kill virally infected cells [3]. In addition, a population of large granular lymphocytes known as CD56^+^ Natural Killer (NK) cells are also frequently anti-viral in nature [4]. Many immune responses are initiated and controlled by the activities of dendritic cells (DC), which are distributed around the body, especially at mucosal surfaces, and which migrate to local lymph nodes upon the detection of pathogens, but which are relatively rare in the normal blood stream. DC develop from a common CD34^+^ haematopoietic precursor in the bone marrow, but can be separated based on cell surface markers and function into myeloid (mDC) and lymphoid/plasmacytoid (pDC) populations [5].

Current detection methods for cells of the immune system include fixation and chemical staining to reveal morphology, which destroys the cells, or more commonly flow cytometry using fluorescently-labeled antibodies, which can potentially alter the behaviour of the cells under investigation. The development of a label-free optical method that would allow further use and manipulation of identified and unaltered immune cells would be beneficial in both research and clinical settings.

Standard Raman spectroscopy represents a powerful optical methodology that can be used to non-invasively generate a chemical fingerprint of a sample, and has been used successfully on both cells and tissues [6,7]. Standard Raman spectroscopy has been used to study immune cells [8,9], and discriminate between cells of the adaptive and innate immune system in the form of lymphocytes and neutrophils respectively [10]. Discrimination of closely related immune cell subsets has not been achieved to date. We have recently shown that Wavelength Modulated Raman Spectroscopy (WMRS) [11] can be an effective enhancement over the standard technique by suppressing the natural luminescent background frequently present in biological samples [12–16] WMRS thus holds the potential to permit specific and sensitive discrimination of the wide variety of cells of the immune system. Whilst WMRS may characterise immune cells isolated from a single individual donor [17], key issues remain with regard to the validity of any study with multiple donors, developing robust laser systems and finally implementing accurate multivariate analysis in such a scenario. To address all three of these aspects, we demonstrate the use of WMRS for the first time on a tunable Ti:Sapphire laser to distinguish between CD4^+^, CD8^+^ T cells and CD56^+^ NK cells. In our work, for the first time, we derive these cells from multiple donors. Finally we also show that WMRS can distinguish pDC and mDC cell populations. This study thus presents a powerful label-free technique for specific immune cell discrimination of closely related cell types.

## Materials and Methods

### Ethics statement

This study was approved by the School of Medicine Ethics Committee, University of St Andrews: project MD6324—Investigation of immune cell behaviour. Samples were obtained after obtaining written informed consent. Participant information sheets and consent forms were also approved by the School Ethics Committee.

### Cell purifications

10 to 30 ml blood samples were collected into heparin Vacutainer tubes from healthy donors. Peripheral blood mononuclear cells (PBMC) were separated on Histopaque (Sigma, Poole UK) and washed in PBS/0.1% bovine serum albumin (BSA) (Sigma) or PBS/0.5% fetal calf serum (FCS), (Life Technologies, Paisley, UK). Cells were isolated using Dynabeads (Life Technologies) untouched human CD4 T cell kit (depleting antibodies comprising anti-CD8, CD14, CD16a, CD16b, CD19, CD36, CD56, CD123 and CD235a), Dynabeads untouched human CD8 T cell kit (depleting antibodies comprising anti-CD4, CD14, CD16a, Cd16b, CD19, CD36, CD56, CD123 and CD235a), Dynabeads untouched human NK cell kit (depleting antibodies comprising anti-CD3, CD14, CD36, HLA Class II, CD123 and CD235a). Dendritic cells were isolated using Miltenyi Biotec (Bisley, UK) MACS plasmacytoid dendritic cell isolation kit II, and MAC myeloid dendritic cell isolation kit (depleting antibodies not specified in the DC isolation kits).

### Flow cytometry

Cells were blocked in 50% PFN buffer (PBS and 2% FCS) and 50% human plasma, then stained with PE-anti human CD4, PE-anti human CD8, PE-anti human CD56, APC-anti human CD303 and APC-anti human CD1c (ebiosciences, Hatfield, UK). Flow cytometry was performed on a Guava easycyte 8HT (Millipore, Hayward, USA) running Guavasoft version 2.5.

### Functional assays

IL-2 Assay: 80,000 CD4^+^ T cells were incubated with or without 0.5 μl Human T-Activator CD3/CD28 Dynabeads (Life Technologies, Paisley, UK) and left at 37°C in a 5% CO_2_ incubator overnight. The supernatant was then assayed using a Human IL-2 ELISA Kit (Life Technologies, Paisley, UK). IFN-γ ELISPOT Assay: IFN-γ was assayed using Human IFN-γ alkaline phosphatase conjugated ELISPOT kit (MABTECH, Nacka Strand, Sweden). 200,000 PBMC and untouched CD8^+^ T cells were incubated with 10 μg/ml of the HLA-A11 restricted Epstein-Barr virus (EBV) peptide AVFDRKSDAK at 37°C in a 5% CO_2_ incubator for 48 hours. CD107a Degranulation Assay: 100,000 NK cells were incubated with or without 10,000 MHC class I deficient 721.221 cells for 6 hours at 37°C in a 5% CO_2_ incubator. After the first hour, 2 μl of FITC conjugated CD107a (ebioscience, Hatfield, UK) was added to samples. Samples were blocked, washed and analysed by flow cytometry as above.

### Raman spectroscopy

A thick quartz slide (25.4 mm x 25.4 mm, 1 mm thickness, SPi Supplies, UK) was used, forming a chamber by placing a vinyl spacer of 80 μm thickness on top. 20 μl of cell suspension in PBS was placed in the well. A second thin quartz slide (25.4 mm x 25.4 mm, 0.15 mm to 0.18 mm thick) was placed on top to form a seal. By inverting the chamber for around 30 minutes, cells settled onto the thinner slide. This obviated any movement caused by optical forces induced by the incident Raman laser. This sample was then placed on the confocal microscope with the thinner slide towards the objective.

Single-cell Raman spectra were recorded using a confocal Raman microscope. The system was equipped with a tunable Ti:Sapphire laser (Spectra-Physics 3900s, wavelength of 785 nm, maximum power 1W) to excite Raman photons which were collected by a monochromator (Shamrock SR-303i, Andor Technology) with a 400 lines/mm grating, blazed at 850 nm, and a deep depletion, back illuminated and thermoelectrically cooled CCD camera (Newton, Andor Technology). A 50x oil immersion objective focussed the laser (Nikon, NA 0.9), delivering in the sample plane a power of 150 mW. A 500 μm confocal aperture produced a confocal cylinder with base radius of 5 μm and height 5.4 μm. By continuously acquiring Raman spectra with a 5 s single acquisition time over a period of 5 minutes, it was confirmed that the laser dosage used does not cause any damage or denaturing to the cells as no variation in the Raman spectra of a single cell was observed during this period of time. Five Raman spectra were collected from each single cell at different excitation wavelengths for a total modulation range of Δλ = 1 nm. The acquisition time for each single spectrum was 5 s. Raman spectra in the region of 600 cm^-1^ to 1800 cm^-1^ were used for subsequent analysis. Raman spectra were collected from 60–80 cells from each cell subset, and from three separate donors. In total 180–240 Raman spectra were collected from each of the following cell subsets: CD4^+^, CD8^+^ and CD56^+^. Some of these Raman spectra were collected on different days to confirm the stability of the system. Fewer cells were recorded for dendritic cell isolations, pDC spectra from 53 cells derived from two donors, and for mDC spectra from 123 cells from three donors.

### Processing the WMRS data

In a first step, each of the five spectra from a single cell was normalised with the total spectral intensity calculated by integrating over all spectral data (Matlab 2014b). This allows to compensate for any power fluctuation in the laser during wavelength modulation. In a second step, principal component analysis (PCA) was used to analyse these normalised five spectra collected from each single cell, with each excitation wavelength step as a parameter, in order to produce a modulated Raman spectrum with essentially all fluorescence background suppressed [15]. This modulated Raman spectra is defined by the first principal component. Within this representation, all standard Raman peaks are indicated by the zero crossing points and the modulated Raman spectrum is similar with a differential spectrum.

### Statistical analysis on Raman data

A parametric student’s T test comparing the location parameter of two independent data samples was applied to the WMRS spectra of any two cell-subsets in order to find the differences between them. Taking into account all the WMRS spectra recorded from immune cells we created a training dataset. We applied PCA to the dataset to reduce dimensionality of modulated Raman spectra. The first 7 principal components were selected as they accounted for the major variance in the dataset. We assessed the ability of this training dataset to distinguish between different cell subsets by using the method of leave-one-out cross-validation (LOOCV), which determines the principal components from the whole data set without one modulated Raman spectrum. This LOOCV spectrum was then classified in the space defined by the principal components using the nearest neighbour algorithm. Correct and incorrect classifications of all cells were then summarised in a confusion matrix. Specificity and sensitivity were estimated for each two cell-subsets.

## Results

### Functional characterisation and flow cytometry of purified cell subsets

**Fig 1:**
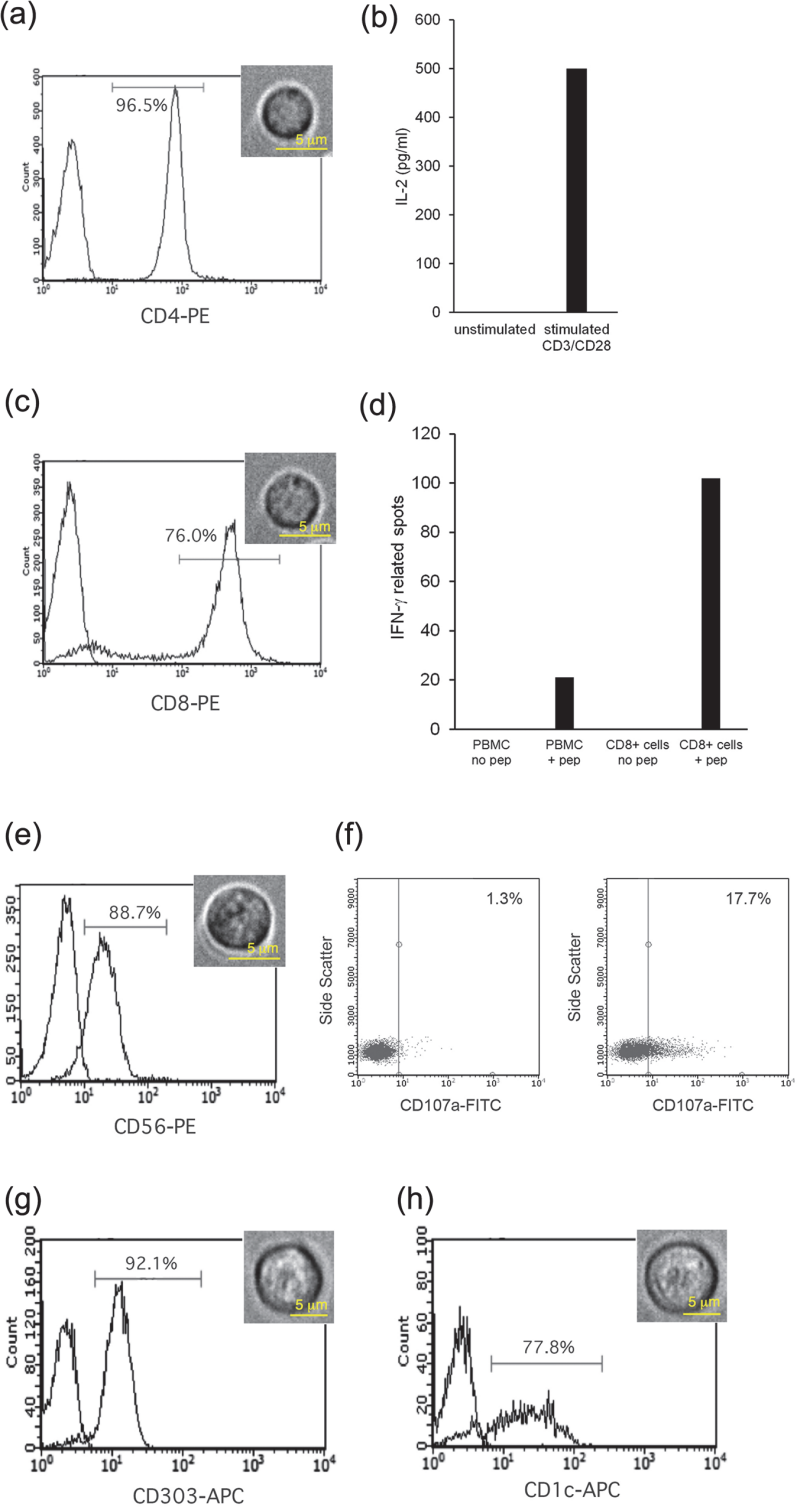
Flow cytometric and functional characterisation of purified cell subsets. (a) CD4 staining of isolated CD4^+^ T cells. (b) IL-2 ELISA of CD4^+^ T cells stimulated with or without anti-CD3/CD28 beads. (c) CD8 staining or isolated CD8^+^ T cells. (d) IFNγ ELISPOT assay of PBMC and purified CD8^+^ T cells incubated with and without EBV derived peptide AVFDRKSDAK. (e) CD56 staining of isolated NK cells. (f) NK cell degranulation assay—CD107a staining of NK cells incubated without (left panel) or with (right panel) MHC class I deficient. 221 cells at a 10:1 effector to target ratio. (g) CD303 staining of isolated pDC. (h) CD1c staining of isolated mDC. The x-axis in each flow cytometry plot indicates fluorescent intensity. The left hand peak in each flow cytometry plot indicates control staining with an irrelevant antibody. Representative white-light microscopy images of each of the purified cell populations used in Raman spectroscopy experiments are also shown.

We purified cells by negative depletion from PBMC, resulting in untouched populations of lymphocytes and DC, to prevent labeling with antibodies that may add to Raman signals, or partially activate the cells under investigation [8]. The isolated cells were analysed for purity by flow cytometry and also tested for biological activity commensurate with their phenotype. CD4^+^ T lymphocytes were obtained at a purity level typically up to 96.5%, and secreted high levels of the cytokine IL-2 in response to incubation with beads coupled with anti-CD3 and—CD28 antibodies (Fig 1A and 1B). CD8^+^ T lymphocytes were obtained at a purity level typically up to 76% (Fig 1C). When stimulated with the Epstein Barr Virus (EBV) peptide AVFDRKSDAK using cells from an individual known to express HLA-A11, which binds this peptide, IFNγ secretion was induced from PBMC and in increased amounts from purified CD8^+^ cells (Fig 1D). CD56^+^ NK cells were obtained at a purity level typically up to 88.7%, and displayed a typical CD56^low^ phenotype (Fig 1E). NK cells are sensitive to the lack of major histocompatibility complex (MHC) class I molecules on target cells, and upon incubation with the HLA class I deficient. 221 cell line, increased expression of CD107a from 1% to 17% was observed, indicating redistribution of CD107a to the cell surface during degranulation leading to target cell lysis (Fig 1F). CD303^+^ plasmacytoid (also known as lymphoid) DC were obtained at purity levels up to 92.1% (Fig 1G) and CD1c^+^ myeloid DC were obtained at purity levels up to 77.8% (Fig 1H). Light microscopy images representative of the purified cell populations are also shown, revealing the CD4^+^ and CD8^+^ T lymphocytes to be small lymphocytes around 7 μm in size (Fig 1A and 1C), the NK cells to be larger at around 9 μm, typical of their historical classification as large granular lymphocytes, and pDC and mDC to be around 9 μm in size.

## Label free characterisation of T cell subsets and NK cells using WMRS

**Fig 2:**
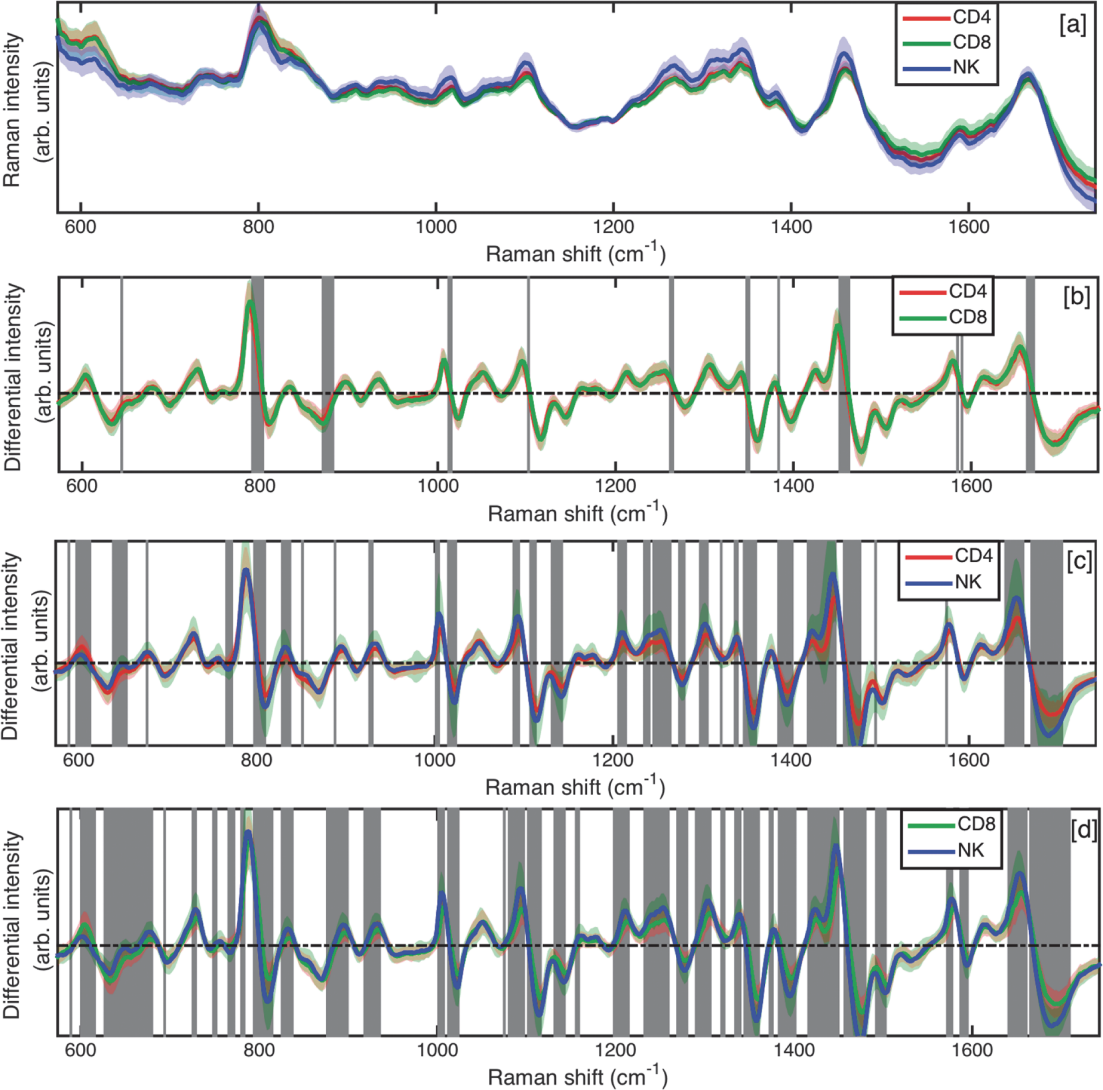
WMRS spectra of purified immune cell subsets. (a) Mean standard Raman spectra of CD4^+^, CD8^+^ and CD56^+^ NK cells. (b)-(d) Pairwise comparison of the WMRS spectra obtained from purified lymphocyte subsets. (b) Mean spectra of CD4^+^ versus CD8^+^ T cells. (c) Mean spectra of CD4^+^ T cells versus CD56^+^ NK cells. (d) Mean spectra of CD8^+^ T cells versus CD56^+^ NK cells. Solid spectra lines represent mean of each subset, with shadow lines representing the standard deviation. Shaded vertical bands indicated regions of significant difference, estimated by student’s T test at level of p<10^–7^.

Single cell Raman spectra in WMRS mode with suppressed fluorescence background were recorded from purified CD4^+^ and CD8^+^ T lymphocytes, and also from CD56^+^ NK cells. Spectra were recorded from between 60 and 80 cells for each of the cell subsets, and from three different donors, resulting in a total of between 180 and 240 Raman spectra for each cell subset overall. Spectra were also recorded over several days to confirm system stability. For comparison, standard Raman spectra collected from the same set of CD4^+^, CD8^+^ and CD56^+^ NK cells are shown in Fig 2A, where a high background can be readily be seen. A pairwise comparison of the WMRS spectra collected from the CD4^+^, CD8^+^ and CD56^+^ cell subsets is shown in Fig 2B–2D. Differential spectra are shown by the mean spectra of each cell subset with their respective standard deviations. Raman bands showing significant differences are highlighted with vertical shading, and were estimated with a student’s T-test at a significance level of p<10^–7^.

**Fig 3:**
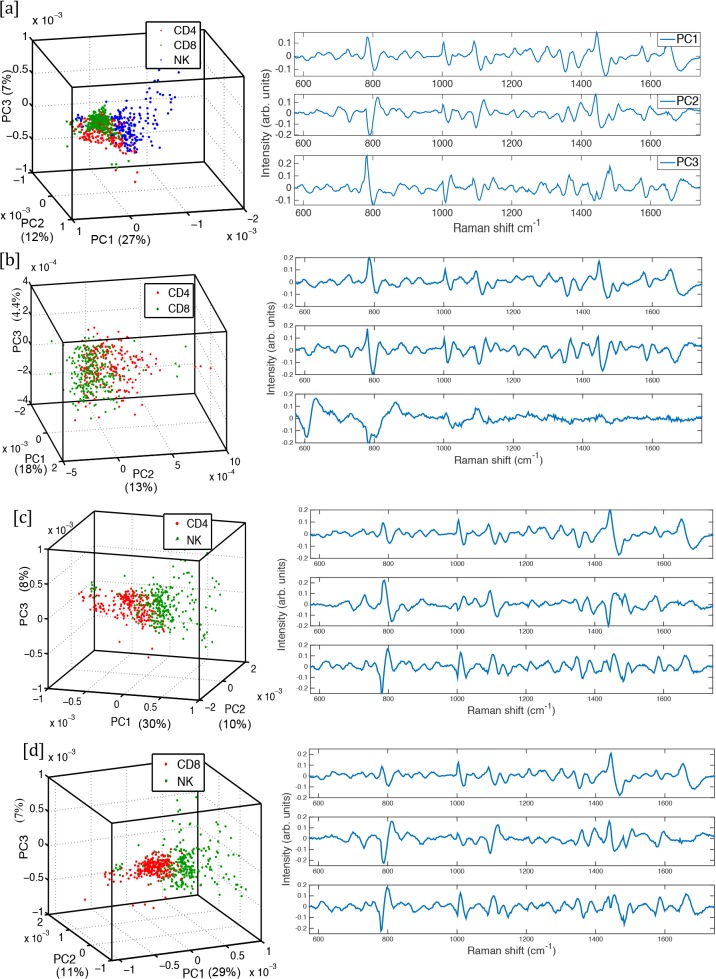
Cluster plots showing the first three principal components for each cell subset isolated from three individuals, with their corresponding first three loadings shown on the right. (a) CD4^+^, CD8^+^ T cells and CD56^+^ NK cells. (b) CD4^+^ and CD8^+^ T cells. (c) CD4^+^ T cells and CD56^+^ NK cells. (d) CD8^+^ T cells and CD56^+^ NK cells. (3D rotating views of these plots are available to view in the supplementary information).

Principal component analysis (PCA) was applied to a training dataset of the cell subsets and the first seven principal components were used to obtain feature reduction of the dataset. As shown in Fig 3, using the first three principal components of each cell subset, in each case there are distinct clusters formed. Thus WMRS identifies distinct fingerprints for each of the CD4^+^, CD8^+^ and CD56^+^ cell subsets.

The efficiency of discrimination of the full dataset using the first seven principal components was then verified using leave-one-out cross validation (LOOCV). The discrimination of CD56^+^ NK cells from CD4^+^ and CD8^+^ T cells yielded a specificity of 93% and 96% respectively and a sensitivity of 92% and 97% respectively. Between CD4^+^ and CD8^+^ T cells the discrimination was lower at 68% specificity and 69% sensitivity, indicating these two closely related cell lineages are more difficult to differentiate between. Using the entire dataset of 638 cells, a confusion matrix was generated (Table 1). Correct predictions located on the diagonal of the matrix indicated good discrimination. Off diagonal numbers indicate classification errors and closely related populations (for example CD4+ and CD8+ T cells). Standard Raman data were also recorded for these same cell subsets. However they did not provide as clear discrimination as WMRS, for example the discrimination of CD56^+^ NK cells from CD4^+^ was lower, with a specificity of 91% and a sensitivity of 90%, and therefore details are not presented here.

**Table 1:** Confusion matrix for CD4^+^, CD8^+^ and CD56^+^ cell subsets.

	Predicted CD4^+^	Predicted CD8^+^	Predicted CD56^+^
Actual CD4^+^	135	84	12
Actual CD8^+^	81	149	1
Actual CD56^+^	24	4	148

The majority of numbers occur on the diagonal indicating good discrimination between the three cells subsets.

### Inter-donor variability

**Fig 4:**
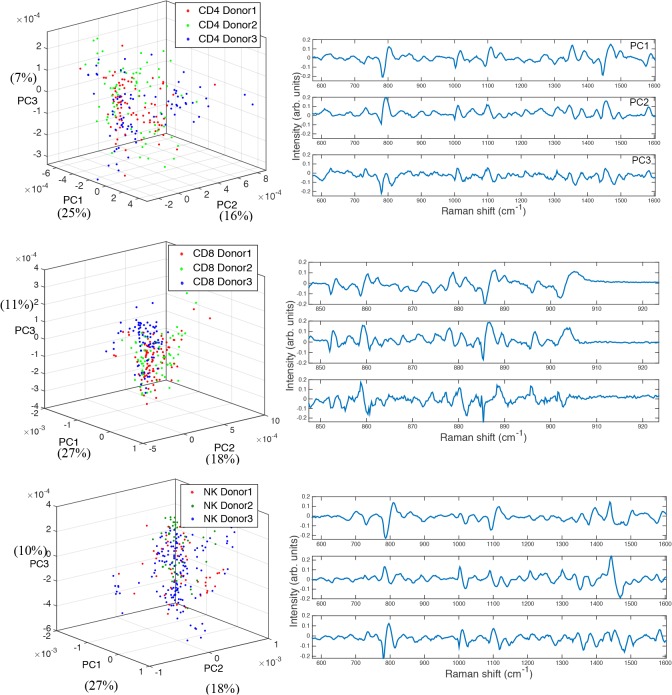
Cluster plots of the first three principal components for each cell type from three donors, with their corresponding first three loadings shown on the right. (a) CD4^+^ T cells. (b) CD8^+^ T cells. (c) CD56^+^ NK cells. (3D rotating views of these plots are available to view in the supplementary information).

Inter-donor variability between the cell subsets was then investigated by analysing all Raman spectra obtained from three different donors. A student’s t-test was performed between Raman signals obtained from each donor with a significance level of p<10^–7^. For each cell subset, no Raman band region showed significant difference among different donors (data not shown). Further, PCA was performed on the dataset for each cell type and the cluster plots of the first three principal components obtained from these three donors are shown in Fig 4. The dataset from three different donors display considerable overlap, confirming that there are no significant differences between the Raman signatures of CD4^+^ T cells from three donors, CD8^+^ T cells from three donors, or CD56^+^ NK cells from three donors. In comparison to a single donor study across all three cell-subsets, the specificity and sensitivity values are marginally higher for a single donor (CD4^+^ vs. CD8^+^: 71% and 69% respectively, CD4^+^ vs. CD56^+^: 97.5% and 96% respectively, and CD8^+^ vs. CD56^+^: 96% and 97.5% respectively), demonstrating the validity of this approach for multiple donors.

### Dendritic cell subset discrimination

**Fig 5:**
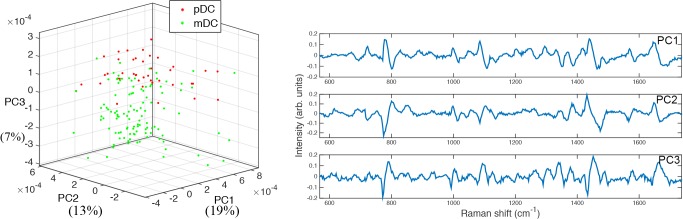
Cluster plot showing the first three principal components for the analysis of pDC and mDC subsets. The corresponding first three loadings are shown on the right.

WMRS was also performed on isolated pDC from two donors and mDC from three donors. As with the lymphocyte cell subsets, PCA revealed the presence of distinct cell clusters, achieving a specificity of 87.7% and sensitivity of 71.1% (Fig 5). Thus WMRS can be used effectively to identify not only the common lymphocyte cell subsets present in blood, but also the more rare blood dendritic cell populations.

## Discussion

The ability to detect non-disruptively, in a completely label-free manner distinct immune cell subsets would be of significance in both *in vitro* and *in vivo* studies of the immune system. With the increased focus and abilities to study cellular behaviour and contents at the single cell level, the necessity of isolating and characterising cells that have not been altered becomes increasingly important. The commonly used techniques of antibody labelling combined with flow cytometry or magnetic bead isolation run the risk of partial modification or activation of the cells, depending on what cell surface marker is being targeted by the antibody. The advantage of a totally optical technique for identification of cells is not only that the cells are unaltered, but that it may also be combined with techniques such as optical tweezing to isolate cells of interest from complex cultures for further analysis such as cytokine-specific rtPCR or full transcriptome analysis by RNA-Seq [18,19].

In this current study, we have used the technique of WMRS, which can significantly diminish the background autofluorescent signal inherent in many biological samples [15], to successfully identify a number of important immune cell subsets, including those found at high frequency in normal human blood, such as CD4^+^ and CD8^+^ T cells, and CD56^+^ NK cells, as well as much rarer immune cell subsets in human blood in the form of CD303^+^ pDC and CD1c^+^ mDC. Of the subsets investigated here the two that proved most difficult to distinguish were CD4^+^ and CD8^+^ T cells. This may reflect their close differentiation lineage origins in the environment of the thymus, where initially double positive CD4^+^CD8^+^ thymocytes undergo a process of thymic education to eventually become either single positive CD4^+^ T cells or single positive CD8^+^ T cells before release into the periphery [20]. Since neither naïve CD4^+^ T cells or CD8^+^ T cells normally contain lytic granules [21], appearing only upon antigen or cytokine stimulation in the latter cells, their inherent difference from granule-containing CD56^+^ NK cells may potentially be attributed to this fact. A future study of primed or activated CD8^+^ T cells, which have synthesized new granules, in comparison to NK cells would therefore be of interest.

Our ability to discriminate between pDC and mDC populations is also of great interest. The pathway for differentiation of the various types of DC is still not fully understood, with a recent report identifying a novel progenitor for pDC [22]. WMRS may thus be an effective technique that could help to further distinguish DC lineages.

A question not addressed in this current study is the identity of the chemical bonds and molecules contributing to the differences in Raman spectra that we have observed for our cell subsets. However, based on published observations and the zero crossings in the measured modulated Raman spectra reported in our study, we can suggest some key areas of difference. Major differences between Raman spectra of CD4^+^ and CD8^+^ are found mainly from C-C twist in tyrosine (around 645 cm^-1^), the O-P-O symmetric stretching (around 800 cm^-1^ and 1097 cm^-1^), symmetric ring breathing mode of phenylalanine (around 1007 cm^-1^), Amide III (around 1259 cm^-1^), polynucleotide chain (around 1345 cm^-1^), thymine/adenine/guanine (around 1378 cm^-1^), CH2 deformation in lipids (around 1455 cm^-1^), adenine/guanine (around 1585 cm^-1^) and amide I (around 1665 cm^-1^). Even more differences were found in the Raman spectra of T cells and NK cells, such as C-C twist in phenylalanine (around 621 cm^-1^), C-S stretching in cysteine (around 671 cm^-1^), adenine ring breathing (around 725 cm^-1^), skeletal C-C stretch in lipids (around 1129 cm^-1^), phenylalanine/tyrosine/C-N stretching (around 1209 cm^-1^) and adenine/amide III (around 1304 cm^-1^) [23–25].

Variability in the WMRS signal between cell types obtained from different donors could be a significant impediment to the application of this technique. Reassuringly, no such variation between donors was found in a previously reported study on neutrophils using standard Raman spectroscopy [10], and similarly we did not detect significant variability in the modulated Raman signals on our isolated lymphocytes or DC.

Our observations lay the foundation for future studies to characterise all the cells of both the innate and adaptive immune systems, both in non-activated and activated states. Furthermore, within each of the major classifications of lymphocytes presented here reside further subsets. For example, the CD4^+^ T cell lineage can be subdivided into at least three further categories, in the form of Th1, Th2 and Th17 cells, characterised by their typical pattern of cytokine secretions. Future studies to determine if WMRS can distinguish between these subsets would be of great potential use, especially for Th17 cells which are associated with a number of disease conditions [26–28].

The cells used in this study are essentially identical in their morphology when isolated from blood. Our WMRS technique thus provides a robust, completely label-free method to identify these closely related cells, and represents a major step forward towards the realisation of a non-destructive, label-free identification technology for cells of the human immune system.

## Supporting Information

S1 Movie CD4^+^ and CD8^+^ T cells, and CD56^+^ NK cells. Cluster plot animation file for the data presented in Figs 3–5, showing the first three principal components for each indicated cell subset.
(AVI)

S2 Movie CD4^+^ and CD8^+^ T cells. Cluster plot animation file for the data presented in Figs 3–5, showing the first three principal components for each indicated cell subset.
(AVI)

S3 Movie CD4+ T cells and CD56^+^ NK cells. Cluster plot animation file for the data presented in Figs 3–5, showing the first three principal components for each indicated cell subset.
(AVI)

S4 Movie CD8^+^ T cells and CD56^+^ NK cells. Cluster plot animation file for the data presented in Figs 3–5, showing the first three principal components for each indicated cell subset.
(AVI)

S5 Movie CD4^+^ T cells from three donors. Cluster plot animation file for the data presented in Figs 3–5, showing the first three principal components for each indicated cell subset.
(AVI)

S6 Movie CD8^+^ T cells from three donors. Cluster plot animation file for the data presented in Figs 3–5, showing the first three principal components for each indicated cell subset.
(AVI)

S7 Movie CD56^+^ NK cells from three donors. Cluster plot animation file for the data presented in Figs 3–5, showing the first three principal components for each indicated cell subset.
(AVI)

S8 Movie pDC and mDC cells. Cluster plot animation file for the data presented in Figs 3–5, showing the first three principal components for each indicated cell subset.
(AVI)
